# Comparative Evaluation of Dental Pulp Tissue Dissolution Ability of Sapindus mukorossi and Sodium Hypochlorite

**DOI:** 10.7759/cureus.51820

**Published:** 2024-01-07

**Authors:** Sriram Kaliamoorthy, Sreeram Rayar, Shanmugapriya SundarRaj, Sugantha Priya Sayeeram, V.V. Premkumar, Sapna C Muddappa, Venkatraman Muthukumaran, Kanmani Raju, Agila Samidorai

**Affiliations:** 1 Department of Dentistry, Vinayaka Mission's Medical College and Hospital, Vinayaka Mission's Research Foundation (DU), Karaikal, IND; 2 Department of Conservative Dentistry and Endodontics, Chettinad Dental College and Research Institute, The Tamil Nadu Dr. M.G.R. Medical University, Chennai, IND; 3 Department of Oral Medicine and Radiology, Government Dental College and Hospital, The Tamil Nadu Dr. M.G.R. Medical University, Pudukkottai, IND; 4 Department of Prosthodontics, Government Dental College and Hospital, The Tamil Nadu Dr. M.G.R. Medical University, Pudukkottai, IND; 5 Department of Conservative Dentistry and Endodontics, SRM Dental College, SRM Institute of Science and Technology, Chennai, IND; 6 Department of Conservative Dentistry and Endodontics, Amrita School of Dentistry, Amrita Vishwa Vidyapeetham, Kochi, IND; 7 Department of Horticulture, School of Agricultural Sciences, Dhanalakshmi Srinivasan University, Trichy, IND; 8 Department of Oral Medicine and Radiology, Chettinad Dental College and Research Institute, The Tamil Nadu Dr. M.G.R. Medical University, Chennai, IND; 9 Department of Periodontics, Chettinad Dental College and Research Institute, The Tamil Nadu Dr. M.G.R. Medical University, Chennai, IND

**Keywords:** endodontic irrigant, sodium hypochlorite, sapindus mukorossi, pulp tissue, : dental

## Abstract

Background

The *Sapindus mukorossi* (SM) extract has been reported to possess antibacterial, antifungal, anti-inflammatory, and antioxidant characteristics. However, there is limited research demonstrating the effectiveness of SM in dissolving dental pulp tissue.

Methods

In an in vitro investigation, pulp tissue samples were extracted from human teeth, collectively weighing 144 mg. These samples were divided equally and activated by manual digital agitation (MDA) or ultrasonic (US) irrigation for three 30-second cycles with a resting period of 45 seconds between each activation. The samples in each group were sub-categorized into a set of three groups based on the treatment received as normal saline (NS), 5.25% sodium hypochlorite (Hypo), or *Sapindus mukorossi *(SM). Statistical tests, including the student t-test and one-way analysis of variance (ANOVA), were employed to compare the mean weight differences among the groups, with a significance level set at p ≤ 0.05 for all comparisons.

Results

The one-way analysis of variance (ANOVA) and independent t-test revealed significant intergroup differences (p<0.05). Turkey’s post hoc analysis indicated significant distinctions, particularly when comparing Hypo with the other two irrigants, namely Hypo-NS (p<0.05) and Hypo-SM (p<0.05) when the MDA method was employed. Considering only the method adopted, the US technique was significantly superior (p=0.04) to the MDA.

Conclusion

*Sapindus mukorossi* (SM) demonstrated efficacy in dissolving pulp tissue but was not as effective as sodium hypochlorite (Hypo) which is the standard agent for root canal irrigation. SM to be used as an alternative to Hypo on clinical grounds needs further validation from research.

## Introduction

The dental pulp, situated within the root canals of human teeth, is safeguarded against mechanical, chemical, and microbial influences by the enamel, dentin, and cementum layers of the tooth. Embedded within the pulp are sensory and autonomic nerve endings, contributing to perception and defensive mechanisms. When faced with microbial intrusion through dentinal tubules and other external stimuli, the dental pulp activates a defensive response. Occasionally, undifferentiated mesenchymal cells within the pulp tissue generate reparative dentin, diminishing permeability to external irritants [1]. The inflammatory reaction of pulp tissue triggered by prolonged external stimuli advances to a state of irreversible pulpitis or pulp necrosis. Dental caries, a chronic infectious ailment affecting dentition, induces irreversible damage to the pulp, impairing its natural reparative capabilities [2]. The microbe's continued invasion towards the apex, coupled with the escalating inflammation of the pulp tissue, extends into the periradicular region via the apical foramen. Ultimately, this leads to inflammation in the periodontal tissue and alveolar bone, requiring prompt intervention to remove the dental pulp tissue and prevent the progression of pulpal disease [3].

The extirpation of dental pulp from the root canals is performed through Root Canal Therapy (RCT). The elimination of dental pulp and the disinfection of the root canal involve both mechanical preparation and chemical irrigation. Research indicates that following the mechanical preparation of root canals, a substantial portion, ranging from 35% to 53%, of the dentinal surface remains untouched by instrumentation. To address this, a chemical irrigant is employed to disinfect the uninstrumented dentinal surface effectively. Chemical irrigants play a crucial role in removing organic debris from the dentinal surface [4].

Chemical irrigants are employed to eliminate smear layers formed during mechanical preparation. Root canal disinfection utilizes various chemical irrigants, including sodium hypochlorite (NaOCl), chlorhexidine, and ethylenediaminetetraacetic acid (EDTA). Among these, sodium hypochlorite is predominantly utilized due to its potent antimicrobial and antibiofilm properties [5].

Sodium hypochlorite (NaOCl) is the most widely employed solution, usually at concentrations ranging from 0.5% to 5.25%. NaOCl presents a high tissue-dissolving capacity and a wide-spectrum antimicrobial activity [5-7].

Prudent handling is essential when using sodium hypochlorite (NaOCl) as a root canal irrigant, as its apical extrusion beyond the apical foramen during Root Canal Therapy (RCT) can lead to post-treatment inflammation and pain [8]. Given the challenges associated with NaOCl, numerous studies have explored the effectiveness of herbal agents as alternatives for root canal irrigation. Investigations have been conducted on herbal irrigants such as neem, German chamomile, Morinda citrifolia, and oregano oil in the realm of endodontics [9]. *Sapindus mukorossi* (SM), commonly known as wash nut, holds a notable role in Ayurvedic medicine and has been employed for addressing diverse conditions such as arthritis, eczema, and gout. The extract derived from SM contains numerous active phytocompounds with inherent surfactant, antibacterial, antifungal, anti-inflammatory, and antioxidant properties. Additionally, the plant extract exhibits antibacterial efficacy against both gram-positive and gram-negative bacteria [10]. Given the benefits associated with this plant extract, the present study aims to evaluate the effectiveness of *Sapindus mukorossi* extract in dissolving dental pulp, comparing its efficacy to that of NaOCl.

## Materials and methods

This in vitro investigation utilized pulp tissue specimens derived from human teeth that were extracted following specific inclusion and exclusion criteria. Permanent teeth without caries, pulpal pathology, or developmental anomalies, necessitating extraction, were included in the study. Teeth with dental caries, pulpal pathology, or developmental anomalies, as confirmed by pre-operative radiographs were excluded from the study. Prior to commencing the research, approval was obtained from the institutional human ethical committee (IHEC-II/0260/22; dated 10th October 2022).

**Figure 1:**
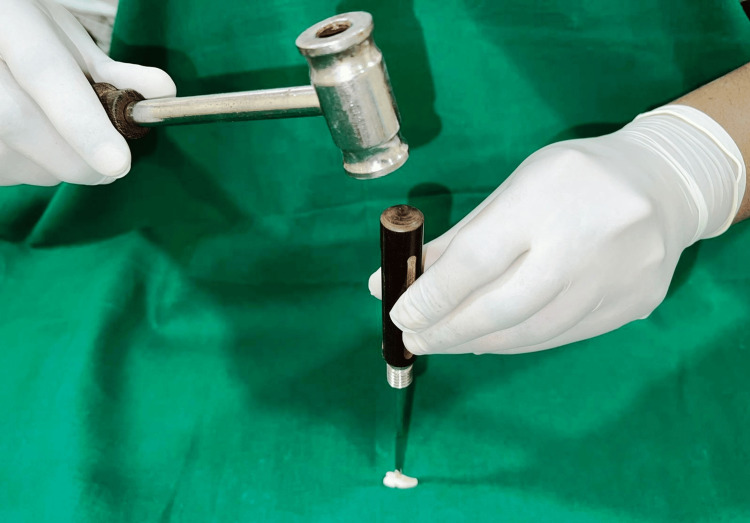
Manual sectioning of teeth using a chisel and mallet

Extracted teeth specimens were sectioned by the creation of longitudinal grooves along the tooth's length using an airotor handpiece. Subsequently, manual splitting was carried out at the middle third of the root, utilizing a chisel and mallet (as shown in Figure 1).

The extraction of pulp tissue from the chamber was carried out meticulously using a tweezer and a number 15 BP blade. Subsequently, the samples underwent a gentle cleansing with distilled water to eliminate debris, followed by blot drying and deep freezing at -80 degrees Celsius until needed. The procured samples were then blended to create a random mix of human pulp tissue. The total weight of this pulp tissue mixture collected was approximately 144 mg. The pulp samples were then randomly divided into a total of six groups, each consisting of three groups with two sub-groups.

**Figure 2:**
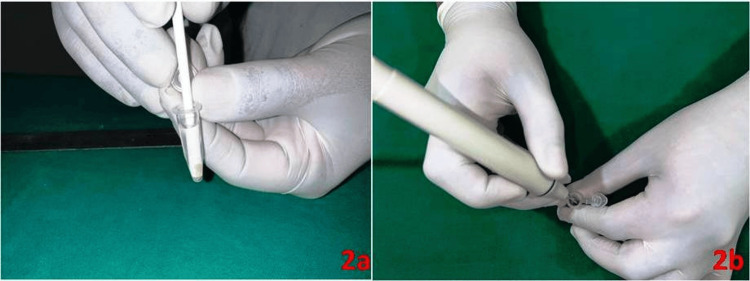
Activation methods used in the study (2a) Manual digital agitation (MDA) and (2b) ultrasonic (US) method.

The first group (NS) received treatment with normal saline OTSUKA Sodium Chloride 0.9% from Otsuka Pharmaceuticals, Ahmedabad, India, serving as a negative control. The second group (Hypo) underwent treatment with 5.25% sodium hypochlorite (NaOCl) from Safe Endo Hypochlor Forte, Gujarat, India, acting as the positive control. The third group (SM) underwent treatment with *Sapindus mukorossi* (concentration of 1-2%), commercially available as 'Reetha liquid extract,' an alcohol-free solvent extracted product using vegetable palm glycerine, devoid of preservatives, gluten, and heavy metals with a shelf life of two years. Subsequently, each group was subdivided into two, and the irrigants used in these subgroups underwent activation through manual digital agitation (MDA), as shown in Figure 2a, and ultrasonic (US) methods, as illustrated in Figure 2b. These subgroups were then labeled as NS-MDA, NS-US, Hypo-MDA, Hypo-US, SM-MDA, and SM-US, resulting in a total of six groups.

Each group comprised 12 individual pulp tissue samples, each weighing 2 mg, and totaling 24 mg per group. Every preweighed pulp tissue sample was subjected to treatment with the respective irrigant in an Eppendorf tube containing 10 ml of the designated irrigant at room temperature. In the MDA and US groups, activation occurred by immersing Gutta-percha (GP) and a Satelac ultrasonic endodontic tip #K25 into the irrigant. Manual activation involved agitating the irrigant using a GP stick. Activation cycles, both in MDA and US methods, consisted of three 30-second cycles with a resting period of 45 seconds between each activation. The total exposure time of the pulp tissue to the irrigant was approximately 4 minutes [11]. Subsequently, the remaining pulp samples were removed, blot dried, and rechecked for their weight.

Consistency was ensured by employing a 2 ml solution and conducting irrigation for standardized 4-minute duration across all samples in the specified groups. A uniform approach was adopted, with a single investigator utilizing a 10 ml syringe for the irrigation process. The assessment of pulp tissue dissolution involved the measurement of pulp tissue weights using an Internal Analytical balance from Sartorius, Goettingen, Germany, both before and after the application of irrigants. Statistical tests were employed to compare the mean differences in weights within each group.

Statistical analysis

The data were collected and processed using statistical software, including R version 4.0.3 and Microsoft Excel (Microsoft® Corp., Redmond, WA, USA). Continuous variables are expressed as mean ± standard deviation (SD), while categorical variables are presented through a frequency table. Inter-group comparisons of means were conducted using the student t-test, paired t-test, and one-way analysis of variance (ANOVA) as necessary. A p-value of ≤ 0.05 indicates statistical significance.

## Results

The present study conducted a comparative analysis of the effectiveness of *Sapindus mukorossi* (SM) in dental pulp tissue dissolution in comparison to 5.25% sodium hypochlorite (NaOCl/Hypo), with normal saline (NS) serving as the control. The evaluation of pulp tissue dissolution involved measuring the mean difference in pulp tissue weight before and after the application of irrigants. Additionally, the manual method (MDA) was juxtaposed with the ultrasonic method (USA). A total of 60 dental pulp tissue samples were categorized into six groups, namely NS, Hypo, and SM, each with a combination of the manual method (MDA) and the ultrasonic method (US). Significance was determined with a p-value of <0.01 using the student t-test or unpaired test, and the intragroup comparisons are detailed in Table 1.

**Table 1:** Intra-group comparison between manual and ultrasonic methods * Significant, ** Highly significant; NS: normal saline; p<0.05 is taken for significance; SM, *Sapindus mukorossi;* Test used: Independent t-test

		Mean	Std. Deviation	Test value	P-value
Normal Saline	Manual	0.167	0.038	-0.761	0.455
	Ultrasonic	0.333	0.065		
Hypo	Manual	0.45	0.066	-8.403	0.0005**
	Ultrasonic	0.70	0.073		
SM	Manual	0.016	0.038	-6.439	0.0005**
	Ultrasonic	0.133	0.049		

Significant distinctions were observed when comparing the three study groups using both the manual method (p<0.0005) and the ultrasonic method (p<0.0005), as detailed in Table 2.

**Table 2:** Inter-group comparison of NS, HYPO and SM using one-way ANOVA * Significant, ** Highly significant; NS, normal saline; p<0.05 is taken for significance; SM, *Sapindus mukorossi*; Test used: ANOVA (one way)

MANUAL			
	Mean & SD	F-value	P-value
NS	0.167 ± 0.038	312.1	0.005**
Hypo	0.45 ± 0.066		
SM	0.016 ± 0.038		
ULTRASONIC			
	Mean & SD	F-Value	P-Value
NS	0.333 ± 0.065	383.9	0.005**
Hypo	0.70 ± 0.073		
SM	0.133 ± 0.049		

The post hoc analysis indicated notable variations, revealing significant differences when comparing Hypo with the other two irrigants, namely Hypo-NS (p<0.0005) and Hypo-SM (p<0.0005), in the manual method. Similar outcomes were observed when employing the ultrasonic method. Regarding SM, significance was established in comparison to NS in the ultrasonic method (p<0.001) but not in the manual method. Refer to Table 3 for detailed information.

**Table 3:** Post Hoc Tukey test following ANOVA * Significant, ** Highly significant; NS, normal saline; p<0.05 is taken for significance; SM, *Sapindus mukorossi*; Test used: Turkey's post hoc-analysis.

	Irrigant	P-value
Manual method	NS	0.0005**
	Hypo	
	Hypo	0.0005**
	SM	
	SM	1.000
	NS	
Ultrasonic method	Irrigant	0.0005**
	NS	
	Hypo	0.0005**
	Hypo	
	SM	0.001**
	SM	
	NS	

The study groups exhibited a significant difference (p=0.04) between the irrigation methods employed. The ultrasonic method appears to outperform the manual method when utilizing any intracanal irrigant. Refer to Table 4 for detailed information.

**Table 4:** Comparison between manual and ultrasonic method regardless of irrigant * Significant, ** Highly significant; NS, normal saline; p<0.05 is taken for significance; SM, *Sapindus mukorossi*; Test used: Independent t-test.

Methods	Mean	Std. Deviation	Test value	P-value
Manual	0.163	0.21	-2.008849	0.048*
Ultrasonic	0.288	0.30		

## Discussion

*Sapindus mukorossi* (SM) is a medicinal plant belonging to the Sapindaceae family and is cultivated in the Indo-Gangetic plain and southern China. It is commonly known by various names such as 'Soap nut,' 'Aritha tree,' or 'Chinese soapberry' [12]. All parts of the SM plant exhibit medicinal or therapeutic attributes. As indicated by Shah et al., the methanol-extracted stem bark of SM has been shown to possess analgesic, anti-inflammatory, and anti-pyretic properties [13]. Additionally, Singh et al. demonstrated the antimicrobial properties of the SM leaf through an in vitro study [14]. The administration of hydroalcoholic extract from SM fruits demonstrated antihyperglycemic and antihyperlipidemic effects in diabetic rats [15].

Dental caries, a prevalent infectious ailment of the oral cavity, is primarily caused by Streptococcus mutans (S. mutans). When left unaddressed, advanced dental caries can lead to root canal infection, with Enterococcus faecalis (E. faecalis) being a frequently identified bacterium in such infections. SM is also utilized in the treatment of oral cavity diseases. The hexane and methanol extracts from SM fruit have demonstrated inhibitory effects against both S. mutans and E. faecalis [16]. The oil extracted from SM seeds exhibited inhibitory properties against periodontal pathogens and effectively mitigated the advancement of periodontitis, leading to a notable reduction in total bone loss in animal models [17].

Root Canal Treatment (RCT), a therapeutic approach for addressing irreversible pulpal infection, incorporates various irrigants to ensure sterile canals following the extraction of dental pulp tissue. Sodium hypochlorite (NaOCl), a commonly employed irrigant for this purpose, poses potential short-term and severe complications if used improperly. Even with cautious and safe application, accidental extrusion into the periapical region may result in pain, localized swelling, burning sensation, tissue necrosis, ecchymosis, and, in extreme cases, airway obstruction, necessitating emergency intervention. Although rare, if not promptly addressed and treated, NaOCl accidents can lead to serious complications [18-20].

Given the drawbacks associated with NaOCl, different herbal irrigants are being explored as alternatives. These herbal irrigants can be categorized according to their properties, encompassing those with antimicrobial, chelating, and pulp-dissolving capabilities. SM is identified as an herbal irrigant specifically known for its pulp tissue-dissolving ability [21]. The current study aimed to evaluate the effectiveness of SM, an herbal irrigant, as a potential alternative.

In the present investigation, it was observed that the application of SM extract on prepared vital pulp tissue led to a noteworthy alteration in the mean pulp weight difference for both Hypo and MS groups. However, no significant change was observed in groups utilizing normal saline (NS) as an irrigant in vitro. A comparable study indicated that SM extracts from various solvents exhibited substantial pulp-dissolving capabilities after a 15-minute treatment, with the methanol-derived SM extract demonstrating superior dissolving capacity (57%). In the current study, commercially available SM oil, free from alcohol, was utilized for treatment. While the study highlighted the significant pulp dissolving capacity of the SM extract, it was found to be inferior to NaOCl, consistent with the findings of the present study. Notably, the study did not assess dissolution capacity concerning different methods of activation [22].

In this study, two different methods of irrigant activation, namely manual and ultrasonic, were compared. The findings indicated that root canal irrigants activated by the ultrasonic method exhibited a higher capacity for pulp dissolution compared to manual activation, regardless of the irrigant used. Other studies that conducted similar comparisons of the ultrasonic and manual activation methods concluded that the ultrasonic approach is more effective for activating root canal irrigants [23, 24]. This superiority is attributed to the ultrasonic activation's efficient removal of pulp remnants and tissue debris, resulting in lower smear layer scores when compared to the manual activation of irrigants [24].

The effectiveness of SM extract is also influenced by the choice of solvent. Comparative analyses revealed that SM extract obtained from ethanol, methanol, and butanol exhibited more potent antimicrobial activity against *P. gingivalis* and *F. nucleatum* in comparison to SM extract derived from distilled water [25]. In a study by Farooq et al., the impact of ethanol extract of SM on the microhardness of human dentin was investigated. The findings affirmed that using SM extract as an irrigant did not significantly alter the hardness of human dentin when compared to 17% EDTA [26].

The in vitro nature of the study design may be a drawback as it does not replicate a clinical scenario. Given the benefits observed with SM extract, further research or clinical trials in vivo should be considered to establish the standardization of SM extract as a potent root canal irrigant.

## Conclusions

*Sapindus mukorossi* (SM) has shown effectiveness in dissolving pulp tissue; however, it falls short of the efficiency achieved by sodium hypochlorite (Hypo), the established agent for root canal irrigation. SM could serve as a potential alternative to Hypo in root canal irrigation, pending validation through clinical trials.
